# Body image construction and mental health levels among college students: a data survey of Chinese university students

**DOI:** 10.3389/fpubh.2023.1268775

**Published:** 2023-10-05

**Authors:** Xing Wang, Chuntian Lu, Long Niu

**Affiliations:** ^1^School of Sports Economics and Management, Xi’an Physical Education University, Xi’an, China; ^2^Department of Sociology, School of Humanities and Social Sciences, Xi’an Jiaotong University, Xi’an, China

**Keywords:** body image, physical exercise, psychological health, social support, college student

## Abstract

**Background:**

With the rapid changes in body image construction brought about by the upgrading of consumption in China, trend-seeking college students are faced with mental health problems brought about by the pursuit of the “ideal body type,” which cannot be ignored. This study aims to explore the relationship between body image construction and mental health among college students. This study utilized data from the Survey on Physical Activity and Mental Health of College Students. A total of 1,192 students were randomly selected as the survey sample, and 1,044 valid samples were obtained. The mean age of the respondents was 19.34 years.

**Methods:**

First, we categorized body image constructs into three categories based on the differences between subjective and objective body image: high acceptance, low acceptance, and consistency. Second, to ensure analytical rigor and minimize potential confounders, we used a generalized propensity score weighting model. Finally, we used a causal mediation framework to investigate the potential causal mechanisms between the independent variable (perceived body image bias) and the dependent variable (mental health) in order to better understand the “net effect.”

**Results:**

(1) There is a significant correlation between college students’ body image perceptual bias and mental health, i.e., the higher the individual’s acceptance of his/her own body image, the higher the level of mental health, and vice versa. (2) Students in humanities and social sciences are more likely to have increased psychological burden due to poor negative body image. (3) In the mediation analysis, although the causal mediating effect of physical exercise was not significant, family and peer support in physical exercise played an important mediating role, especially the influence of peers was more significant.

**Conclusion:**

The construction of body image is a double-edged sword that can either promote positive individual development or lead to self-depreciation. Creating a positive climate for physical activity has a positive impact on college students’ mental health compared to participation in physical activity behaviors. While improving students’ media literacy on college campuses, it is important to enhance adaptive guidance to promote their physical and mental health and personal development.

## Introduction

In China, an intriguing phenomenon has emerged among college students, manifesting in their various online “photo-sharing” activities that have left netizens astounded, proclaiming that “going to college is akin to undergoing plastic surgery.” The stark contrast between their initial “outdated” appearance upon entering college and their subsequent “fashionable” transformation upon graduation has sparked fervent discussions and inspired countless imitations among internet users. As the notion that “good looks and figure equate to justice” becomes the guiding principle for constructing body image, college students’ approach to body management increasingly leans toward external evaluations. This includes the widespread dissemination of a series of body image ideals, notably the pursuit of an “ideally thin” physique, which resonates deeply within the college student community. A survey conducted in China revealed that nearly 70% of college students have engaged in weight loss behaviors in their pursuit of a satisfactory body shape (1). However, as the definition of a “satisfactory body shape” undergoes changes, their actions may also undergo transformations.

In response to this phenomenon, scholars have conducted a series of studies, yielding two contrasting conclusions. Scholars adopting a negative attitude argue that as college students excessively pursue weight loss and an ideal body shape, their satisfaction with their own body image experiences a downward trajectory, acting as a catalyst for “psychological weight gain” and resulting in adverse effects on mental health (2, 3). Beneath the surface of these unrealistic weight loss efforts lies the irrational convergence of body image construction, potentially harboring more negative factors (4). Furthermore, the pursuit of an ideal body shape has led to the proliferation of unhealthy methods of body shaping among young people. These detrimental practices have become a sort of “magic potion” for young individuals, enticing them with the illusion of swiftly attaining their desired physique (5). Conversely, scholars with a positive outlook approach the issue from the vantage point of symbolic interactionism, perceiving the body as a “symbolic medium.” They contend that the pursuit of an ideal body shape can facilitate individuals’ integration into higher social circles and grant them access to a wider array of external resources. They maintain that the process of striving for an ideal body shape represents a positive state of being, generating uplifting psychological stimuli and fostering the expansion of one’s social networks when met with external affirmation (6–8).

The exploration of the relationship between body image construction and mental health levels in college students has become a pressing issue that warrants in-depth investigation. In today’s modern society, the process of shaping body image is intricately intertwined within various micro-level social interactions, and it is far from being static. Individuals experience significant variations in social comparisons and psychological changes across different environments. Moreover, within the same environment, disparities in family resources and the subsequent development of information-gathering abilities greatly shape the differentiated process of body image construction. As a highly educated segment of society, college students aspire to align themselves with societal trends in order to enhance their overall self-image and information acquisition quality, especially in our fast-paced society (9). Simultaneously, as they progress in their personal development, they become more attuned to the vast array of information available, leading to heightened sensitivity toward body image. Therefore, it is crucial to explore how college students perceive their own bodies and the subsequent impact of body image on their mental well-being. This study aims to investigate the cognitive biases in body image perception among college students, utilizing a survey conducted on the physical and mental well-being of Chinese college students. Through this analysis, we aim to shed light on the influence of body image on their mental health levels and provide practical recommendations.

## Literature review and research hypotheses

The concept of “body image” was first introduced by Paul Schilder in the 1920s, and it has since become a critical area of study. Schilder defined body image as an individual’s perception of their physical and psychological attributes, as well as their attitudes toward their own body characteristics (10). However, body image is not simply a matter of objective body shape; it is a multidimensional concept that encompasses perception, emotion, cognition, and behavior (11). In the process of constructing body image, individuals are often influenced by external evaluations. Chinese scholar Hong Chen describes body image as a psychological portrayal of an individual’s body, including their cognitive understanding, attitudes toward physical and psychological functions, and the impact on their behavior (12, 13). Body image is subjective, transitioning from external evaluations to individual actions, and it also involves emotional evaluation (11, 14). What sets college students apart from other youth groups is their ability and willingness to take proactive measures in shaping their body image. They actively link their body image with their personal emotions and gradually integrate it into their self-concept. Moreover, the relatively open campus environment and the desire to conform to societal trends make having a desirable body shape a central aspect of college students’ self-perception (9, 15).

With the emergence of consumer culture and liberalism on college campuses, the plasticity of the body undergoes changes as self-perception evolves, leading to a more stable body shaping (16, 17). This stable body shaping includes college students’ self-perception of their own bodies, which serves as a vital conduit between the “self” and the external world (18). However, social comparisons in different contexts can result in cognitive biases. The external environment’s influence on body image interacts closely with self-perception, ultimately alleviating the distress caused by societal pressures through the fulfillment of self-body image (2, 19). Xin et al. found that the micro-system environment of college, while providing some insulation from external pressures and reducing pre-existing disparities, highlights the value of self-body perception as individuals integrate into the college micro-social environment (13, 20). From the outset, they strive to cultivate a positive body image, translating their knowledge into action to shape their ideal physique—a promising beginning for college students (4, 12, 21). In the open campus environment, college students’ body image construction reflects their psychological adjustment. However, it is important to be cautious of body image influenced by cultural norms. External influences continuously mold individuals, leading to a partial loss of self-image awareness (5, 13). In other words, the impact of the external environment on college students becomes increasingly subtle, resulting in cognitive biases in body image perception and a relatively stringent body ideal (15, 22, 23). As such, college students’ self-perception of their bodies holds particular significance for their mental well-being (3).

In today’s society, both in the physical world and online, the visible presence of obesity plays a significant role in shaping one’s self-image. However, obesity is not merely a physical state; it has become laden with social connotations. Consumer institutions cleverly exploit the plasticity and malleability of the body, using body-centric marketing to reshape people’s perceptions of obesity and transcend its superficial implications. This external construction of the obese image not only places immense pressure on individuals but also fosters a demanding body image environment, prompting individuals to distance themselves from the negative associations of obesity and sometimes resorting to unhealthy weight loss strategies. Moreover, obesity is stigmatized, becoming a symbol of societal discrimination and prejudice. Shockingly, studies reveal that 65% of overweight and obese adults and 77% of overweight adolescents are subjected to negative and derogatory portrayals, with some even being depicted as intellectually inferior or lacking self-control (5). This stigma extends beyond individuals to educational institutions, where 28% of teachers consider obesity to be the worst thing that can happen to a person, and even parents may inadvertently provide less support to overweight children (24). In the digital age, the negative impact of obesity on body image is further exacerbated, hindering meaningful social interactions and perpetuating the stigmatization of those affected (15, 24). Consequently, obesity has become a primary target of body image “shaming,” with widespread dissemination of derogatory portrayals. Obese individuals constantly face distorted body perceptions, the judgment of others, discrimination, and the burden of societal stigma, all of which erode their sense of bodily autonomy and self-worth (8, 24, 25).

Even when their weight is within the normal range, more than 60% of women choose to engage in weight loss efforts (26, 27). Research suggests that as the consumer culture surrounding the body becomes increasingly prevalent, women willingly conform to external evaluations in order to enhance their life opportunities, thereby reinforcing these evaluations in the process. This strong desire for body shaping leads college students to unilaterally pursue an ideal body shape, often resorting to unhealthy weight loss methods in their pursuit of thinness as the epitome of beauty (26, 27). When individuals are influenced by distorted body perceptions in their external environment, they further reinforce negative body image perceptions in social interactions (16, 28, 29). Negative body image perceptions become the primary trigger for body shape anxiety among college students, to the extent that deviations in body image perceptions lead to significant self-deprecation regarding their own body image (30). The cognitive habits associated with negative body image also have a profound impact on self-esteem and tendencies toward unhealthy weight loss, serving as a psychological inertia that deepens college students’ dissatisfaction with their bodies. If college students hold preconceived misconceptions, it will inevitably lead to increased anxiety and unease in their college lives (25). However, from another perspective, college students with a higher level of body image acceptance are more likely to alleviate body shape anxiety. For instance, research indicates that within the college campus, the prevalence of diverse cultures weakens the one-sided “thinness as beauty” body image construction, thereby reducing the likelihood of individuals experiencing body shape anxiety (27). Based on these findings, this study proposes hypothesis 1.

*H1*: Lower body image acceptance will have a negative impact on the mental health of college students. Conversely, higher body image acceptance will have a positive effect on mental health.

It is noteworthy that prior studies have aimed to investigate the influence of body image perception distortions on mental health by considering social divisions and structural heterogeneity, including factors such as gender, class, and even regional disparities (1, 2, 10, 22). While significant interventions from external environments have been observed, these structural factors often emerge naturally, disregarding the potential impact of individual choices. Professional choices, for college students, are not only personal decisions but also comprehensive selections influenced by various conditions, including familial and societal relationships, thereby being subject to diverse structural factors. Consequently, for college students, professional choices replace structural differences, becoming more nuanced and multifaceted forms of heterogeneity. For instance, research has revealed that students in STEM fields appear to have lower self-imposed body image standards, whereas those pursuing humanities and social sciences exhibit relatively positive attitudes (31–33). Within the college student population, the division of majors holds significant representativeness. Building upon these insights, hypothesis 1a is proposed:

*H1a*: There are professional differences in the relationship between body image cognitive biases and mental health levels.

Bourdieu posits that body image construction is a pivotal factor in attaining status and differentiation, serving as a pathway to acquiring bodily capital (6). This process has often been simplified as “perception-action-outcome” in previous studies. While college students come from diverse backgrounds, once they step foot on the university campus, their lives undergo a transformation, presenting a relatively uniform living experience. The previous academic competition is diminished, making room for new forms of competition. Notably, the construction of a body image that aligns with the university culture becomes an integral aspect. Drawing on existing research, physical exercise emerges as a key element in shaping body image (34, 35). It not only serves as a tool for self-transformation but also as a means of showcasing and obtaining symbolic value (36–38). This study specifically focuses on the impact of body image perception distortions on mental health, with physical exercise being the primary avenue for body management. It not only translates natural bodily symbols into discernible symbolic orders but also gives rise to biased body expectations (6). Consequently, physical exercise not only positively influences body image construction but also significantly contributes to promoting psychological well-being. In fact, it is even recognized as an adjunct therapeutic approach for mental health conditions. As body image construction influences physical exercise, it subsequently generates positive effects on mental health (11, 19, 35). Thus, we propose the following hypothesis.

*H2*: Physical exercise has a mediating effect on the relationship between body image construction and mental health levels.

In addition, the construction of body image is a complex interplay of both objective and subjective factors, representing individuals’ perception and development of body value within the realm of body management (36, 39). Body capital holds significant value for individuals in their social interactions and facilitates the transition of capital types. The relationship between body perception and the external environment is robust, with social capital exerting substantial influence on the construction of body image (40, 41). Consequently, the construction of body image becomes a process through which individuals transition from body capital to other forms of capital. However, this process is influenced by individual tastes, habits, and social positions, leading to diverse impacts. Moreover, the social comparison of body image brings about changes in social capital, which, in turn, affects the mechanism of “body image-mental health.” Notably, the influence stemming from social capital also significantly impacts psychological well-being. Based on these insights, we propose hypothesis 3: the influence of the sports atmosphere.

*H3*: Social support has a mediating effect on the relationship between body image and mental health.

### Data sources

This survey employed online methods such as WeChat and utilized random sampling. It was conducted in September 2021 in the provinces of Shaanxi, Hunan, and Gansu. The survey targeted undergraduate students (first, second, third, and fourth year) and some graduate students from Xi’an Jiaotong University, Xi’an University of Electronic Science and Technology, Shaanxi Normal University, Xi’an University of Technology, Xi’an Technological University, Tianshui Normal University, and Huaihua College. The selected sample covers a wide range of school categories and majors, including universities involved in the “985 Project,” “211 Project,” local key universities, and regular undergraduate institutions. The majors include science and engineering, economics and management, humanities, agriculture, medicine, law, and foreign languages, among others. The sample structure of the survey is reasonably balanced. The survey collected information on students’ individual characteristics, physical activities, mental and physical health, and social interactions. A total of 1,200 questionnaires were distributed, with 1,192 returned. Ultimately, 1,044 valid questionnaires were obtained, resulting in an effective response rate of 87%.

## Variable selection

### Dependent variable

The dependent variable in this study is the level of mental health among college students. We used the Center for Epidemiologic Studies Depression Scale (CES-D) to measure the level of mental health. To ensure rigor, internal consistency and validity of the questionnaire were tested during the revision process.

First, we examined the reliability of the survey data using Cronbach’s alpha coefficient, which resulted in 0.88 > 0.7, indicating high reliability. The Kaiser-Meyer-Olkin (KMO) measure was used to assess the validity of the questionnaire, and the result was 0.899 > 0.80. Additionally, the Bartlett’s sphericity test (X2 = 80.66, *p* < 0.05) confirmed good structural validity of the questionnaire.

The questionnaire covers 10 questions related to the frequency of experiences in the past 2 weeks, such as “frequency of loss of interest or pleasure in activities,” “frequency of feeling down, depressed, or hopeless,” “frequency of difficulty sleeping, waking up, or excessive sleepiness,” “frequency of feeling tired or having little energy,” “frequency of poor appetite or overeating,” “frequency of feeling dissatisfied with oneself,” “frequency of difficulty concentrating,” “frequency of moving or speaking slowly or being restless,” “frequency of thoughts of death or self-harm,” and so on. These questions are measured on a Likert scale ranging from 1 to 5. We reverse code and sum the responses from low to high intensity, resulting in a mental health score ranging from 0 to 100. A higher score indicates a higher level of mental health.

### Independent variable

This study focuses on the cognitive bias of body image among college students. This variable is composed of subjective perception of obesity and objective body shape. Subjective overweight refers to a situation where the objective body shape is thin, but the subjective perception is overweight, indicating a lower acceptance of body image. Subjective underweight refers to a situation where the objective body shape is overweight, but the subjective perception is underweight, indicating a higher acceptance of body image. The difference between subjective and objective body image is the cognitive bias of body image.

I in the specific operation, BMI is used as the operational variable to measure the objective body shape, following the BMI indicators for Chinese adults. This classification is primarily based on the recommendations of the Working Group on Obesity in China (WGOC) (42). BMI < 18.5 is categorized as “underweight” and assigned a value of 1; 18.5 ≤ BMI ≤ 23.9 is categorized as “normal” and assigned a value of 2; BMI ≥ 24 is categorized as “overweight” and assigned a value of 3.

Second, in order to understand individuals’ subjective perception of their bodies, we used the question “How do you perceive your own body shape?” for measurement. The scale originated from the Chinese General Social Survey (CGSS). To better align with the actual experiences of college students and facilitate their responses, we modified the CGSS question “How do you perceive your current physical health status?” to “How do you perceive your current body shape?” The response options are coded as follows: 1 = “very thin,” 2 = “a bit thin,” 3 = “neither fat nor thin,” 4 = “a bit fat,” 5 = “very fat.” Similarly, the responses are recoded as follows: “very thin” and “a bit thin” are categorized as underweight and assigned a value of 1, “neither fat nor thin” is categorized as normal and assigned a value of 2, “a bit fat” and “very fat” are categorized as overweight and assigned a value of 3.

Finally, a contingency table is used for analysis. The samples in the lower left part of the diagonal in the contingency table indicate higher acceptance of body image and are assigned a value of 1; the samples on the diagonal indicate cognitive consistency and are assigned a value of 2; the samples in the upper right part of the diagonal indicate lower acceptance and are assigned a value of 3.

### Mediating variables (physical activity)

Physical activity (PA) is one of the mediating variables in this study. PA is measured using the International Physical Activity Questionnaire (IPAQ) (short form). The questionnaire consists of 7 questions designed to assess physical activity levels based on categories of walking, moderate, and high-intensity activities. During the data processing, the outliers and truncation principles of the questionnaire are strictly followed, as well as the weights assigned to different intensities of physical activity, to calculate the energy expenditure value (MET) for each student’s PA. MET is used to reflect the level of physical activity among college students, with higher numerical values indicating higher intensity of physical activity. The duration and frequency of reported exercise are also taken into account.

To align the MET variable with a normal distribution, a natural logarithm transformation is used to create a continuous variable that follows a normal distribution. Additionally, to further enhance the mediating effect of physical activity, the three continuous variables are categorized as high, medium, and low, forming three groups of continuous variables.

### Mediating variables (social support)

One of the mediating variables in this study is social support. Drawing from Berkman and Cantor’s categorization of social support (33), social support for college students is divided into two aspects: family support and peer support. In this questionnaire, we utilized the following questions: “My father or male family members encourage me to participate in sports,” “My father or male family members provide guidance for my physical exercises,” “My father or male family members provide material support for my physical exercises,” “My father or male family members provide technical support for my physical exercises,” “My mother or female family members encourage me to participate in sports,” “My mother or female family members provide guidance or encouragement for my physical exercises,” “My mother or female family members provide material support for my physical exercises,” “My mother or female family members provide technical support for my physical exercises.” These questions were scored on a scale of 1 to 5, ranging from “never” to “always.” In the data analysis process, these scores were summed to create a continuous variable, ranging from 0 to 40, with higher scores indicating stronger family support.

Similarly, we utilized the same set of questions, replacing “father” or “mother” with “peers” or “classmates.” These questions also generated a continuous variable ranging from 0 to 40.

### Control variables

This study includes 7 variables: gender, age, grade level, school type, household registration, family socioeconomic status, and self-rated health status. Gender is coded as female = 1, male = 0. School type is categorized as national key university = 3, provincial key university = 2, provincial regular university = 1. Household registration is coded as rural = 0, urban = 1. Family socioeconomic status is categorized as lower = 1, lower-middle = 2, middle = 3, upper-middle = 4, upper = 5. Self-rated health is rated as very poor = 1, poor = 2, fair = 3, good = 4, very good = 5. Additionally, we have included the square of age.

### Analysis strategy

To accurately investigate the relationship between virtual body image and levels of psychological well-being, it is essential to address the potential issue of selection bias between the two. Selection bias arises when individuals have different probabilities of entering different processing levels (21). Previous research has shown that distorted body image perception may be associated with various factors, and these confounding variables can also impact levels of psychological well-being. For example, individuals who frequently use electronic devices are more likely to experience distorted body image perception, and the use of electronic devices itself can have an influence on psychological well-being (42). Therefore, simply comparing the levels of psychological well-being among groups with different perceptual biases makes it challenging to determine whether the observed differences are due to body image perception or the distinct characteristics of these groups with different body image perceptions. The interference of these confounding variables makes it difficult to observe the “net effect” of body image perception bias on levels of psychological well-being. To establish a more compelling causal relationship between virtual body image and psychological well-being, it is necessary to control for these confounding factors in research.

The focus of this study is on the perception of body image, which is categorized into three groups. To ensure the validity of our research, we employ a generalized propensity score weighting approach. This method, as suggested by Guo and Fraser (17), involves incorporating confounding variables into a multinomial logistic regression model to calculate the generalized propensity scores for each individual. These scores are then used to determine individual weights, allowing us to represent the broader population accurately. Following the adjustment for basic sociodemographic characteristics, we utilize a weighted logistic regression model to examine the relationship between educational attainment and levels of general trust.

In our analysis, we aim to focus exclusively on the net effects, employing a causal mediation analysis framework to investigate the causal relationship between the independent and dependent variables. Causal mediation analysis operates within a counterfactual framework, allowing us to capture statistically significant causal relationships (21). Furthermore, this approach offers flexibility in terms of the types of mediator and outcome variables considered, accommodating both continuous and categorical mediators and outcomes. It also accommodates multi-category treatment variables, making it applicable to our study’s multi-category treatment variable—acceptance of overweight body image. Additionally, when examining the mediation effect of a specific mediator variable, we include other mediator variables in the model predicting the outcome variable. This control helps to mitigate the influence of other mediator pathways, preventing confounding and interference from other mediator factors, and enables us to examine the net effect of the mediator variable.

## Results

### Descriptive statistics

Filling in the missing values was done using multiple imputation, as shown in Table 1. Descriptive statistics revealed the following: (1) The average level of psychological health among students in the humanities and social sciences is lower than students in other STEM majors. (2) Among students in the humanities and social sciences, the proportion of those with lower body image acceptance is 22.84%, while the proportion of those with higher body acceptance is 11.20%. In non-humanities and social sciences students, this proportion has changed, with the proportion of those with lower body image acceptance decreasing to 16.90% and the proportion of those with higher body acceptance increasing to 14.95%. Preliminary findings suggest a certain connection between body image perception bias and psychological health. Comparatively, humanities and social science majors have stricter requirements for their body image, with a much higher proportion of students having lower body acceptance compared to other majors. This may be related to the nature of the majors. Research has shown that students choosing humanities and social science majors tend to have a more subjective and intuitive cognition, while STEM students tend to be more objective.

**Table 1:** Factor analysis of items of health.

Variable	Entire sample			Humanities and social sciences major		Non-humanities and social sciences major		*p*
	*N* (%)	Mean	Range	*N* (%)	Mean	*N* (%)	Mean	
Mental health	1,044	76.26	0–100	482	75.56	562	79.80	0.542
Low-intensity exercise	999	6.07	Logarithm	463	6.11	536	6.04	0.032
High-intensity exercise	885	5.80	Logarithm	376	5.91	509	5.72	0.000
Total physical Activity	992	4.32	Logarithm	450	4.03	542	4.67	0.000
Family status	1,044	2.42	1–5	482	2.46	562	2.40	0.000
Parental education	1,044	12.02	6–19	482	11.76	562	12.24	0.000
Grade	1,044	1.82	1–7	482	2.32	562	1.40	0.004
Age	1,044	19.34	17–45	482	20.01	562	18.76	0.253
Body image	1,044	1.56	1–3	482	1.49	562	1.61	0.029
Low acceptance	234 (22.41)			139 (28.8)		95 (16.9)		
Consistency	672 (64.37)			289 (59.9)		383 (68.1)		
High acceptance	138 (13.22)			54 (11.2)		84 (14.9)		
Family support	1,044	19.06	0–40	482	19.39	562	18.76	0.000
Peer Support	1,044	17.07	0–40	482	17.05	562	17.09	0.000

In addition, Table 2 demonstrates the effects of body image differences on mental health and the effects of heterogeneous outcomes across disciplinary majors. In Model 2, the coefficient of disciplinary majors is significantly positive, indicating that students majoring in non-humanities and social sciences have higher levels of mental health. In Model 3, the regression coefficients remained significantly positive when the interaction term between body image differences and disciplinary specialization was added, indicating a positive moderating effect of disciplinary specialization on the effects of body image differences on mental health. Specifically, students in humanities and social sciences are more likely to have increased psychological burden due to poor negative body image, thus validating the hypothesis of H1a.

**Table 2:** The impact of cognitive bias on mental health.

	Model 1	Model 2	Model 3
High acceptance		7.808^***^ (1.41)	8.521^***^ (1.66)
Low acceptance		−7.567^***^ (2.26)	−8.563^***^ (2.42)
Professional category		4.677^***^ (1.21)	2.524^**^ (2.95)
Physical cognitive bias * professional category			1.392^*^ (1.77)
Control variable	Controlled	Controlled	Controlled
*N*	1,044	1,044	1,044

*p < 0.1, **p < 0.05 (standard errors in parentheses).

### Generalized propensity score estimation and weighted processing

Table 3 presents the results of the multinomial logistic regression model predicting the generalized propensity scores. Among the findings, students with higher socioeconomic status and higher grade levels have higher body image requirements, while rural students have a lower probability of being satisfied with their body image. As age increases, college students’ level of non-acceptance of their own body image shows a U-shaped trend, with a decrease followed by an increase. Since we are not specifically interested in the effects of specific confounding variables, we will not provide specific interpretations of the regression coefficients here.

**Table 3:** Multinomial logistic regression results predicting individual body image acceptance propensity scores.

	High acceptance VS consistency	Low acceptance VS consistency
Urban and rural (rural = 0)	−0.271* (0.16)	0.233 (0.28)
Age	0.312 (0.33)	−0.875** (0.35)
Age squared	−0.010 (0.01)	0.014** (0.01)
Family economic status	0.180* (0.10)	−0.373** (0.17)
Number of children	−0.124 (0.15)	0.227 (0.26)
Type of school	−0.263** (0.12)	−0.007 (0.20)
Type of major (Social Science = 0)	0.113 (0.17)	0.085 (0.26)
Gender (male = 0)	0.259 (0.16)	−0.420* (0.25)
Grade	0.245** (0.10)	0.062 (0.18)
Constant terms	−1.937 (3.77)	10.835** (4.72)
Sample size	1,041	

*p < 0.1, **p < 0.05 (standard errors in parentheses).

To suppress the occurrence of covariate imbalance in the matching process, we adopted a weighted approach. The results show that after weighted processing, the distribution of covariates between the treatment and control groups is generally balanced. The propensity score weighting method largely eliminates the covariate imbalance and corrects for selection bias.

After obtaining the propensity scores, we can explore the net effects of body image perception on mental health levels using a generalized weighted approach, according to the analytical strategy. Table 3 presents the regression results after weighted adjustment. Compared to college students with unbiased body image perception, those who are dissatisfied and have difficulty accepting their own bodies are more likely to experience negative effects on their mental health. On the other hand, college students with higher body satisfaction tend to have a positive impact on their mental health levels. The average difference in mental health levels between individuals who accept or do not accept their body image is 10.49. Additionally, through robust estimation, the results remain stable, indicating that individuals with unbiased body perception have an average mental health level of 74.22. After adjusting with generalized propensity score weighting, the impact of body perception on mental health levels increases, suggesting that some confounding factors may have interfered with the relationship between body image perception and mental health levels. The use of propensity score weighting is meaningful.

### Mediation analysis

Table 4 reports the causal mediation effects of different levels of exercise intensity energy expenditure on the relationship between body image perception and mental health levels. The top half of the table presents the linear regression results predicting the mediation variables using the dependent variable, body image perception, for low-intensity, moderate-intensity, and high-intensity exercise expenditure.

**Table 4:** Causal mediation analysis of the effects of body image perception on psychological well-being (Part 1).

	Low intensity		Medium intensity		High intensity	
	Predict mediation variables	Predictor outcome variables	Predict mediation variables	Predictor outcome variables	Predict mediation variables	Predictor outcome variables
**Reference group (unbiased)**						
High	129.386*	3.731***	30.180	3.731***	70.110	3.731***
Low	−48.468	−4.727*	−194.828***	−4.727*	619.279***	−4.727*
Mediation		<0.001		0.005		0.002
Other mediation		YES		YES		YES
Control variables		YES		YES		YES
Intercept	1024.28	97.92***	−951.740	97.92***	1024.280	97.92***
Sample size	1,041					

*p* < 0.1, **p < 0.05, ***p < 0.01 (standard errors in parentheses).

Individuals with lower levels of self-body image acceptance are more likely to engage in moderate to high-intensity physical exercise, while the effect is not significant for low-intensity exercise. Conversely, individuals with higher levels of self-body image acceptance are more inclined to participate in low-intensity physical exercise. In other words, individuals with lower body image acceptance are more willing to engage in moderate to high-intensity physical exercise, while relaxation of body management and willingness to exercise are not prominent, even if they do exercise, they prefer low-intensity workouts. The columns representing the expected outcome variables show the mediation effects of different types of physical exercise on mental health levels, predicted by body image perception, while controlling for all other mediator variables.

The results of the causal mediation analysis also indicate that the impact of body image perception on college students’ mental health levels is primarily a direct effect (ADE), with limited mediation effects through physical exercise. However, college students with stricter body management still have a certain proportion of improvement in mental health levels through moderate to high-intensity physical exercise (Table 5).

**Table 5:** Causal mediation analysis of the effects of body image perception on psychological well-being (Part 2).

Causal mediation effect	High VS consistency	Low VS consistency	Higher VS consistency	Low VS consistency	Higher VS consistency	Low VS consistency
ACME	0.057	0.059	0.132	0.33	0.166	0.44
ADE	5.54	7.83	5.47	7.55	5.44	7.44
Total effect	5.6	7.89	5.61	7.89	5.61	7.89
Proportion of mediation effect	1.02%	0.76%	2.3%	4.1%	2.9%	5.6%

(1) The computation results for the evaluation parameters of the mediating effects were obtained through 1,000 iterations of the quasi-Bayesian Monte Carlo approximation simulation. (2) The “Proportion of Mediation Effect” reported in the table is calculated by dividing the “Average Causal Mediation Effect” (ACME) by the “Total Effect.” The numerical values may have slight differences compared to the results obtained from calculating ACME and total effect directly in the table. This discrepancy is due to the decrease in numerical precision caused by rounding.

Table 6 reports the mediation effects of family support and peer support in the relationship between body image and mental health levels. The table presents the mediation effects of family support and peer support on the relationship between body image perception and mental health levels. The results show that, after controlling for other mediator variables and control variables, both family support and peer support have significant mediation effects.

**Table 6:** Causal mediation analysis of the effects of body image perception on psychological well-being (Part 3).

	Family support		Peer support	
	Predict mediation variables	Predictor outcome variables	Predict mediation variables	Predictor outcome variables
**Reference group (unbiased)**				
High	1.906***	3.731***	2.025***	3.731***
Low	−1.707*	−4.727*	−2.903***	−4.727*
mediation		0.199***		0.123*
Other mediation		YES		YES
Intercept	−7.465***	96.867***	−1.895***	96.867***
Sample size	1,041			

*p < 0.1, ***p < 0.01 (standard errors in parentheses).

Table 7 presents the causal mediation effects. It can be observed that a supportive exercise environment can influence individuals who are part of it, even if they are not directly involved in the exercise. For college students with different levels of self-body image acceptance, the proportion of mediation effects from family support is 11.72%, and for those with different levels of non-acceptance, it is 10.41%. The mediation effects from peer support are 12.15% for acceptance and 13.23% for non-acceptance of body image. This also suggests that, after entering college, a supportive exercise environment is more likely to impact individuals’ mental health levels, and having family resources that support exercise helps mitigate external influences and improve mental health levels.

**Table 7:** Causal mediation analysis of the effects of body image perception on psychological well-being (Part 4).

Causal mediation effect	High VS consistency	Low VS consistency	High VS consistency	Low VS consistency
ACME	0.66***	0.82***	0.68***	1.04***
ADE	4.96***	0.76***	4.93***	6.83***
Total effect	5.61***	7.88***	5.62***	7.88***
Proportion of mediation effect	11.72%	10.41%	12.15%	13.23%

(1) The computation results for the evaluation parameters of the mediating effects were obtained through 1,000 iterations of the quasi-Bayesian Monte Carlo approximation simulation. (2) The “Proportion of Mediation Effect” reported in the table is calculated by dividing the “Average Causal Mediation Effect” (ACME) by the “Total Effect.” The numerical values may have slight differences compared to the results obtained from calculating ACME and total effect directly in the table. This discrepancy is due to the decrease in numerical precision caused by rounding.

## Discussion

Body image plays a pivotal role in comprehensive sex education, and guiding and helping college students to construct a positive body image is of great practical significance to their healthy physical and mental development. This paper classifies the construction of college students’ body image from both objective and subjective levels, and in doing so, explores the relationship between body image cognitive bias and mental health, as well as the potential mechanisms that may exist. The following conclusions were drawn: (1) Through the weighted analysis of the propensity values in Table 8, it was found that college students’ deviation from reality and pursuit of extreme body image would have a more serious negative impact on their mental health. On the contrary, having a more positive attitude toward their body image would have a positive impact on their mental health. At the same time, more demanding body image constructs had a greater negative impact on mental health levels. This finding was subsequently confirmed indirectly in Tables 4, 6. (2) In the moderated effects model in Table 2, we found that students majoring in humanities and social sciences were more sensitive to their body image compared to students majoring in other categories. (3) Through Tables 5, 7, we found that while the causal mediating effect through physical activity was not significant, family and peer support for physical activity played an important mediating role, and the influence of peers in particular was more significant.

**Table 8:** Weighted propensity analysis of the predictive effect of college students’ body image on psychological well-being.

Variables	Before weighting	After weighting
Reference group (unbiased): 74.22		
Low acceptance	−5.234** (2.21)	−5.974*** (2.29)
High acceptance	4.395*** (1.19)	4.514*** (1.19)
Control variables	YES	YES
Constant terms	95.072*** (23.07)	82.538* (44.09)
R2	0.075	0.113
Sample size	1,041	

**p < 0.05, ***p < 0.01 (standard errors in parentheses).

Specifically, Conclusion 1 demonstrates that the distorted construction of body image is a major factor influencing the psychological well-being of university students. This finding aligns with the majority of research conducted in Western societies (7, 11, 24). However, the model construction and explanatory paths in this study differ from previous approaches. Existing studies based on samples from Western countries have predominantly approached the topic from either a psychological or sociological perspective, highlighting distinct differences. The psychological perspective represents an endogenous viewpoint, focusing on subjective body image as the unit of analysis and examining historical, societal, and cultural factors as facets of psychological influences. For example, Cash argues that body image is a multidimensional psychological experience that extends beyond mere appearance (43). On the other hand, the sociological perspective reflects an exogenous viewpoint, considering body image as part of social interactions and its associations with factors such as race, skin color, and regional boundaries. For instance, Bourdieu views the body as a form of “bodily capital” shaped by external environments (6), while Goffman suggests that the body is a tool for social interactions (44). The key distinction between them lies in Bourdieu’s perspective, where bodily capital is determined by an individual’s external circumstances, while Goffman believes that body image is subject to change as social interactions evolve. These studies often attempt to examine subjective or objective aspects of body construction, relying on a stable social context.

However, we believe that this fixed or single path of interpretation may be more applicable to European and American societies and not to China. The reason for this is that China has changed dramatically over the past 40 years of reform and opening up, while European and American societies are relatively stable. Chinese universities, for example, are becoming more and more open and diversified, with both “universities without walls” and “universities at will” existing in the schools we surveyed, and the population of Chinese universities becoming richer and richer, with the student body having changed from the initial “university without walls” to “university at will.” The student body has evolved from an “elite” to a “public.” Therefore, it is difficult to examine the real situation of Chinese university students from a single dimension in such an open and changing external environment. Therefore, we attempted to construct new variables of subjective body image (psychological factors) and objective body image (social factors) to further clarify the relationship between college students’ perceived body image bias and mental health. Based on this, the explanation we give for the first result comes from two aspects.

Based on this, we offer two explanations for the first result. Firstly, the cultural aspect. In China, “fat” and “thin” are the primary factors in assessing body image. In university campuses, being “ideally thin” represents a desirable physique. This contrasts sharply with Western societies. For instance, Stanford University and others have found that Western cultural influences shape the perception of body image, with many males aspiring to a “muscular” physique as their ideal and believing that females prefer muscular men (45). The pursuit of muscularity leads to higher levels of physical exercise participation among Western university students compared to those in mainland China. Secondly, the cognitive aspect. The rise of consumer culture has provided various body techniques for shaping the image of being overweight. University students in China do not need to engage in physically demanding activities that require significant time and effort to shape their bodies; instead, they can rely on clothing, appearance, and balanced diets as relatively flexible and effortless means to achieve their desired body image. This differs significantly from the emphasis on muscularity in Western campuses. In short, Chinese university students prioritize “looking thin” rather than “being healthy thin.” This viewpoint is indirectly supported by a survey conducted by China Youth Network in 2021. The survey revealed that with the increasing openness of Chinese university campuses and the accelerated dissemination of body-related information through the internet, university students have become one of the main consumer groups in the realm of body image. Among those students attempting weight loss, 62.45% do not have good exercise habits (1), making their weight loss endeavors more likely to falter.

Furthermore, our research on professional heterogeneity has also been validated. Despite the limitation of sample size, we categorized the professions into two major groups and observed that the differences among professions significantly moderate the impact of body image distortion on psychological well-being. Previous studies conducted in China have suggested that different professions possess distinct knowledge systems, which consequently contribute to diverse constructions of body image (46). Moreover, further investigation revealed that humanities and social science majors amplify the negative psychological levels associated with low body image acceptance. We believe that this conclusion may also be attributed to the strong job orientation of university majors in China. For students in humanities and social sciences, their work primarily involves complex social contexts. Therefore, cultivating a body image that aligns with current trends would facilitate their professional endeavors.

Finally, the underlying mechanisms differ from previous studies. In prior research, physical exercise was believed to exert positive effects, whereas in our study, this mechanism was replaced by the social support derived from physical exercise. Many previous studies touted physical exercise as a panacea, and various investigations on mental health emphasized the importance of physical exercise. However, they overlooked the fact that physical exercise actions often require alignment with good exercise habits (45), which in turn necessitate inclusive social support as a safeguard. We believe that the lack of significant mediating effects of physical exercise in our study is related to the absence of good exercise habits and an exercise-friendly atmosphere among Chinese university students. We attempt to provide two explanations: firstly, the parents of Chinese university students are products of an era of relative material scarcity, and with the improvement in material conditions, they have placed more emphasis on improving living standards in their offspring’s investment, rather than considering physical exercise as a priority (30). Secondly, Chinese students face intense academic competition and perceive physical exercise as a hindrance to studying, with long-term guarantees being lacking (36). Consequently, the lack of a conducive exercise environment makes it difficult for university students to develop exercise habits. This aligns with what we observed in the mediating effects, where physical exercise did not emerge as an underlying mechanism, which also corresponds to the current research conducted by Chinese scholars. For instance, Chinese scholars utilizing data from surveys conducted between 1985 and 2005 found that Chinese university students increasingly rejected the idea of an overweight body shape, while simultaneously experiencing varying degrees of decline in their physical fitness (47).

The limitations of this study stem mainly from the average effects produced by quantitative research. On one hand, the lack of support from longitudinal data makes it difficult to observe the changing trends in college students’ body image construction. On the other hand, the absence of corresponding interviews hinders further verification of the reliability of the conclusions.

## Conclusion

Based on the above conclusions, this paper argues that the construction of body image and mental health level of college students can not only rely on schools and students, but also need the whole society to create a “healthy and beautiful” standard. Secondly, we should strengthen the educational norms to guide college students to build a healthy and positive body image. Educational institutions can provide relevant health education programs to help students develop correct body image concepts, and provide more appropriate guidance and support based on professional differences. Second, media literacy education should be strengthened to enhance college students’ ability to recognize media content and reflect on it. College students should learn to rationally assess the body image presented in the media and establish positive values and evaluation standards. Finally, school education should emphasize its guiding role and actively guide students to form correct health and esthetic views. Physical exercise can be an effective intervention to help improve the mental health of college students who have cognitive biases in body image. Through these measures, we can promote the physical and mental health of college students and help them better adapt to social life and personal development.

## Data availability statement

The datasets presented in this article are not readily available because for academic research only. Requests to access the datasets should be directed to niulong@xatu.edu.cn.

## Author contributions

XW: Conceptualization, Data curation, Writing – original draft. CL: Formal analysis, Writing – review & editing. LN: Conceptualization, Writing – review & editing.
